# A Case of Parturition, with Its Complications, &c

**Published:** 1855-09

**Authors:** E. F. Knott

**Affiliations:** Griffin, Spalding County, Georgia


ARTICLE II.—*A case of Parturition, with its complications, viz: Procidentia Vaginæ, Procidentia Uteri, Rupture of the Uterus, and Rigidity of the Os Uteri, with successful termination of the labor by forceps, to both mother and child.* By E. F. KNOTT, M. D., of Griffin, Spalding County, Georgia.

Having been spoken to by Mr. Joseph P. Manly, of this county, to attend a negro woman, Jane, aged about twenty-five years, in parturition with her first child, as he expected some difficulty in her case, from the fact of her having labored under Procidentia Uteri previous to her conception, on Monday evening, July 23d, I was summoned to attend her. On arrival, I learned from the old servant midwife of Mr. M. that she was attacked about 11 o'clock, A. M., with facial Neuralgia, which gave way under the use of warm teas, and about 2 o'clock she was taken with the usual labor pains, (as Aunt Winney termed them,) which grew stronger as the evening approached. Upon examination, I found an unusual puffiness, as I supposed, of the vagina, but with as much dilatation of the os uteri as usual at that stage of labor, and her pains were regular but of a feeble character, no doubt from an enervated state of the functions of the uterus from previous disease, as referred to above. I learned that her bowels had not been acted on, although she had taken a full dose of Ol Ricini. I immediately ordered a repetition of the dose, which, not acting, was followed by a large dose of Ol Ricini and fifteen drops Tinct. Opii. All not acting, I ordered an enema of gruel and Ol Ricini, which acted promptly and thoroughly a time or two. During the latter part of the night I found that she had Procidentia, which I supposed to be of the uterus, and asked Mr. Manley for consultation. He immediately called my worthy and esteemed friend, Dr. John R. Clark, who lived within one mile, and soon came to my aid. After stating to him the progress and management of the case, and my impression as to its being Procidentia Uteri, he made an examination and corroborated my opinion that it might be Procidentia Uteri. In the course of our further interview and consultation, I suggested to him the possibility, and even probability, of its being Procidentia Vaginæ; whereupon he made, as well as myself, a more minute and careful examination, and detected clearly the os uteri, as before stated, as much dilated as usual at that stage of labor, and found it to be Procidentia Vaginæ. As her pains were weak and did not seem to accomplish much, we thought it was advisable to use the secale cornutum, which was given in infusion until afternoon, but with little apparent effect.

Dr. Clark remained with me until late in the afternoon, having been absent a few hours about noon, and we were careful to support the fallen vagina as well as possible in its proper place. Soon after Dr. Clark left she had pretty strong pains, which lasted at short intervals until 8 o'clock, P. M., and appearing to acquire new impetus at every onset, I really hoped the labor might be terminated that night; but all at once, about 8 o'clock, the pains ceased, and she rested very well, with the exception of occasional transient pains at longer intervals, as though performed by a uterus enervated from some occult cause. It is proper to state here that the pressure of the fœtal head against the pubis was unusually great, (it being the third presentation of Dewees,) and this may serve to account to some extent for what was discovered afterwards to have occurred. Having rested well, as before stated, until the latter part of the night, when the pains became strong and forcible, I discovered, upon examination, that not only the head of the cl ild, but the uterus, had reached the os externum, and as there seemed to be more rigidity of the os uteri than was proper, I ordered a pediluvium, and endeavored as much as possible to promote dilatation of the os uteri. The labor advanced, but the uterus descended still with the child. While endeavoring to give such assistance as I deem proper in such cases, and tracing the os as well as uterus generally, (having amounted by this time to a procidentia,) I detected a hairy tumor, and at first supposed it to be a portion of the labia and external parts of the patient in a tumid and congested state; but, having traced it more particularly, I satisfied myself it could not be that, and called for a candle, when, to my surprise, I found it to be a puffed tumor of the integuments of the child's head, making its way through a rent in the uterus of about three inches in length, transverse, and about where the cervix and body unite. I immediately asked for my consulting physician, Dr. Clark, who was sent for in great haste. I proceeded forthwith to change the position of the child, and cause it to engage in the proper channel (the mouth of the womb) which I succeeded in doing very promptly. The messenger returning, announced Dr. Clark's departure from home for Griffin, a distance of six miles, before he arrived. I discovered a firm and unyielding contraction of the os uteri upon the child's head, and an irresistible tendency in the uterus to descend at every onset of pain, while the vertex of the child's head seemed to press with unusual force directly against the lacerated portion of the uterus. Knowing that this labor must terminate some way before I could send to Griffin or elsewhere for consultation, (that being the nearest point to any physician,) and no farther dilatation having taken place, which I ascertained by endeavoring to pass the digital finger between the os uteri and the head of the child, I determined to proceed to conduct the labor to as favorable termination as possible. I immediately bled her thirty ounces, and applied cloths wrung out in hot water to the abdomen and vulva, and sent to Dr. Clark's for his instruments. As soon as they arrived, which was in a few minutes, I thought sufficient time had elapsed for the os uteri to yield, and apprehending great danger of the whole os and cervix uteri being torn off by the forcible and repeated contractions, I proceeded, after lubricating the forceps well, cautiously to introduce them, and having them well adapted to the fœtal head and locked, I succeeded by gentle pressure and traction of the blades, aided by the contractions of the womb, in extracting a fœtus, weighing from nine to ten pounds, in a state of asphyxia, which I succeeded in resuscitating by inflating the lungs, employing warm bath, counterirritants, and allowing the circulation between mother and child to be kept up by not dividing the funis. As soon as the child breathed, I proceeded to give such aid as was prudent in facilitating the expulsion of the placenta, which was done with ease. I divided the funis, handed the child to the midwife, and, having applied some Balsam Copaiva and Olive Oil to the lacerated portion of the uterus, I returned it to its proper place; applied a compress saturated with Balsam Copaiva, Olive Oil, and the white of an egg, to the internal organs and abdomen; applied a bandage around the abdomen, and enjoined the most perfect rest and quietude.

On the next day I visited the case; found her comfortable; ordered Castor Oil to be given the following day, and general directions as to cleanliness, strict regimen, and a wash of tepid water and sweet milk to be thrown up the vagina, as a detergent, three times daily, and a decoction of red oak bark to be used immediately after it as an antiseptic, together with such attention to the breasts as was necessary to keep them soft, as well as general directions to keep the bowels open, with the injunction that if things did not go on well I was to be notified instantly.

I have heard from her every day or two since, and had direct intelligence from her to-day, being the fourteenth from delivery, and nothing was wrong. She has a most abundant secretion of milk, and as fine and healthy a child as ever was seen.

I felt it to be my duty to give this succinct history of this case without any comments, hoping that it may in some way prove useful to the profession, and elicit something more valuable than I could confer in the way of contributions by commenting upon it.

Earnestly wishing your most sanguine expectations may be realized, both in your collegiate and journalizing capacity, in disseminating useful and truthful knowledge to the profession, and sincerely wishing the triumphant success of medical science, in which I have an ever-abiding confidence, and in whose fields, although thickly beset with thorns, I delight to range, and know that the more we know of its resources the more fully we become satisfied that they are abundant, glorious and transcendent.

